# Biodegradation of pollutants by exoelectrogenic bacteria is not always performed extracellularly

**DOI:** 10.1111/1462-2920.15942

**Published:** 2022-02-23

**Authors:** Lars J. C. Jeuken

**Affiliations:** ^1^ Leiden Institute of Chemistry Leiden University PO Box 9502, 2300RA, Leiden the Netherlands

Exoelectrogens like bacteria from the *Geobacter* and *Shewanella* species have the ability to transfer electrons extracellularly to minerals for redox balancing of fermentation or to respire on extracellular electron acceptors (Shi *et al*., 2016; Lovley and Holmes, 2022). Extracellular electron exchange is also used between microbes in multi‐species biofilms in anaerobic digestion. Over the last two decades, this exquisite ability of nature to transfer electrons extracellularly has seen a spurge of research into potential new applications. Replacing extracellular electron acceptors with macroscopic electrodes creates microbial fuel cells to convert the oxidation of organics from, for instance, wastewater into electricity (Logan *et al*., 2019). In a reversed direction of electron transfer (i.e. ‘inwards’ rather than ‘outwards’), microbes gather energy from extracellular electron donors, such as Fe(II)‐bearing minerals. This has important consequences to the economy as it enhances biocorrosion, but the same ability is currently being developed for applications in electrobiosynthesis. Biofilms on conductive materials can be ‘fed’ by applying electric potentials to drive synthesis. For some bacteria such as *Shewanella oneidensis*, it has been discovered that their natural ‘outward’ electron transfer direction can be reversed (Ross *et al*., 2011), significantly widening the number of microbes that can be engineered for electrobiosynthesis.

More recently, exoelectrogens, as well as non‐exoelectrogens, have been coupled to light‐harvesting nanomaterials, mainly CdS quantum dots, creating semi‐artificial photosynthetic biohybrids (Sakimoto *et al*., 2016; Wang *et al*., 2019; Martins *et al*., 2021). Such biohybrids could in principle be engineered to use light energy to drive either outward and inward electron transfer (Piper *et al*., 2021). So far, the focus has mainly been on photobioelectrosynthesis, where light‐driven electron transfer into microbes is utilized for the synthesis of organic materials, ammonia or hydrogen.

Early on in these studies, an important model exoelectrogen, *Shewanella oneindensis* MR‐1, was developed for application in bioremediation (Marshall *et al*., 2006; Shi *et al*., 2016). *S. oneindensis* MR‐1 is a dissimilatory metal ion‐reducing bacterium with extreme diverse respiratory capabilities. Pollutants such chromium and radioactive uranium are reduced by *S. oneindensis* MR‐1 from the soluble Cr(VI) and U(VI), to the insoluble Cr(IV) and U(IV), thereby preventing leaching from the polluted grounds. Key proteins from the metal‐reducing (Mtr) pathway were subsequently found to also be responsible for exoelectrogenic capabilities of *S. oneidensis* MR‐1 (Marshall *et al*., 2006). Especially the protein complex MtrCAB was found to transfer electrons across the outer membrane from the periplasm to the extracellular environment. Following on from this early work in metal reduction, it was shown that *S. oneidensis* MR‐1 could reductively bleach a range of azo dye pollutants and the Mtr pathway was often identified to contribute to the rate of decolouration (Watanabe *et al*., 2009; Liu *et al*., 2016).

In spite of the inspiring capability of *S. oneidensis* MR‐1 for extracellular electron transfer, it should not be forgotten that many reduction processes take place in the periplasm of Gram‐negative bacteria. Furthermore, the degradation of chemicals and pollutants in general is often due to chemical processes in the cytoplasm. In work by Zhu *et al*. (2022), reported in this issue of *Environmental Microbiology*, it is indeed observed that nitroaromatic compounds and other pollutants are not necessary reduced outside the bacteria. In particular, they show that the inner‐membrane protein CymA reduces 2,4‐dichloronitrobenzene (DNCB).

Zhu *et al*. took three different approaches to indicate the role of CymA. First, they show that the reduction of DNCB in *S. oneidensis* MR‐1 is not perturbed in Δ*mtrABCDEF*Δ*omcA* mutants, but it is moderated in Δ*cymA* mutants. Δ*mtrABCDEF*Δ*omcA* mutants lack the most important outer membrane cytochromes required for extracellular electron transfer. The fact that this mutant shows the same rate of DNCB removal clearly indicates that extracellular electron transfer does not play an important role in this particular process. Then, they show that expression of a soluble form of CymA in *Escherichia coli* leads to an enhancement in reduction of DNCB in *E. coli*. To express the soluble form of CymA in the periplasm of *E. coli*, the authors make use of a previously constructed chimera of a periplasmic maltose‐binding protein (MBP) and a CymA construct that misses the N‐terminal transmembrane helix, creating a soluble, periplasmic MBP‐Cym_sol_ (Londer *et al*., 2008; Firer‐Sherwood *et al*., 2011). In their third approach, the authors purified MBP‐CymA_sol_ and using protein‐film electrochemistry on meso ITO electrodes, they show that CymA can directly reduce DNCB. Combined, these approaches indicate that CymA is capable of reducing DNCB and thus might contribute to the direct reduction of DNCB in *S. oneidensis* MR‐1. Importantly, however, is that DNCB is still degraded in Δ*cymA* mutants of *S. oneidensis* MR‐1, indicating that multiple mechanisms contribute to the biodegradation of this nitroaromatic compound.

**Fig. 1:**
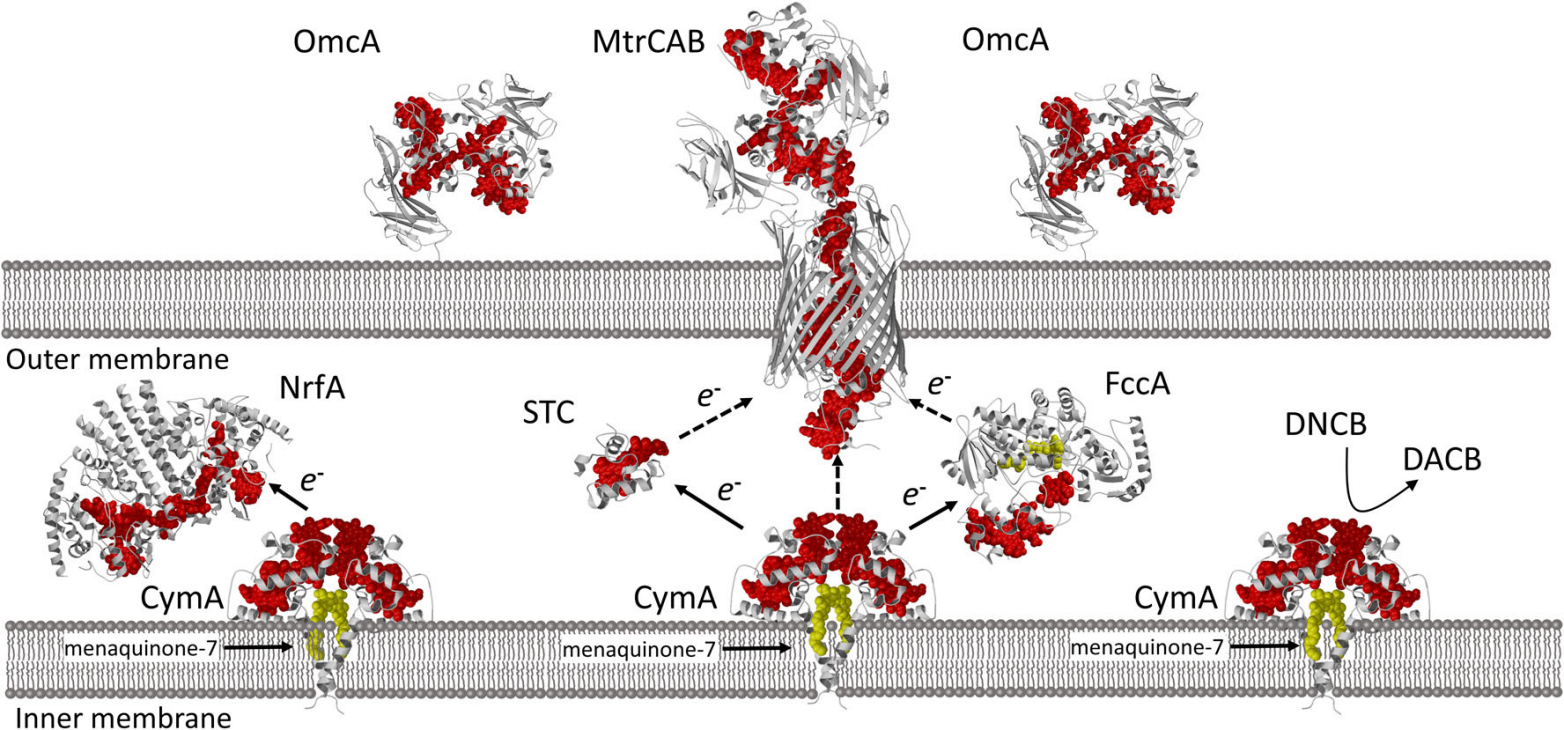
A schematic representation of the periplasm of *S. oneidensis* MR‐1 with a number of reductases for which structures from *Shewanella* species are available (MtrCAB PDB:6r2q; OmcA PDB:4lmh; NrfA: 6p73; STC PDB:1m1p and FccA PDB: 1qjd). CymA is based on the NrfH structure from *Desulfovibrio vulgaris* (PDB:2j7A).

A role of CymA in the biodegradation of DNCB is interesting as CymA is a key electron transfer hub in *S. oneidensis* MR‐1 (Marritt *et al*., 2012a). CymA is a menaquinone reductase, passing electrons from menaquinol‐7 in the inner membrane to a range of periplasmic reductases. Key work by Myers and Reid in the late nineties and early noughties showed that Δ*cymA* mutants lost the ability to respire on a range of terminal electron acceptors, including iron(III), nitrate, fumarate, and manganese(IV), DMSO and nitrite (Myers and Myers, 1997; Schwalb *et al*., 2003). CymA can thus transfers electrons to a nitrate reductase (NapDAGHB), a DMSO reductase (DmsABEF), a nitrite reductase (NrfA), a fumarate reductase (FccA), as well as a small tetraheme cytochrome (STC; Fig. 1). NMR experiments showed that the interaction between STC and CymA is transient (Alves *et al*., 2015), and it is likely true for other CymA‐protein interactions as well. CymA is also key in transferring electrons to the above‐mentioned MtrCAB, although there is a considerable plasticity in the electron transfer pathway and it is unclear how electrons are transferred from CymA to MtrCAB (Fonseca *et al*., 2019). The finding by Zhu *et al*. that CymA might now have a direct role in bioremediation of nitroaromatic compounds adds another function to this already fascinating electron hub.

The molecular structure of CymA has not yet been elucidated. However, the structure of a homologue, NrfH from *Desulfovibrio vulgaris*, is known. NrfH is part of a multiheme NrfH_2_A_4_ complex, which is a menaquinol:nitrite oxidoreductase. Identical to CymA, NrfH oxidizes menaquinol in the inner membrane (McMillan *et al*., 2012). Unlike CymA, however, NrfH transfers electrons only to its partner protein NrfA within a quaternary NrfH_2_A_4_ complex. Spectroscopic and voltametric analysis of CymA confirms that CymA, like NrfH, has four c‐type hemes, one high‐spin heme and three low spin hemes (Marritt *et al*., 2012b). Based on its homology with NrfH, it is likely that CymA has at least three of the four hemes localized close or at its surface. If we assume that CymA does not have an evolution‐based lock‐and‐key binding site for nitroaromatic substrates, there are still ample opportunities for CymA to reduce nitroaromatic compounds at its surface.
